# Un-sweetening the deal: targeting glucose metabolism in brain arteriovenous malformations

**DOI:** 10.1038/s44321-026-00384-x

**Published:** 2026-02-18

**Authors:** Martina Rudnicki, Tara L Haas

**Affiliations:** 1 UCL Institute of Ophthalmology, London, EC1V 9EL UK; 2 York University Faculty of Health, Toronto, M3J 1P3 ON Canada

**Keywords:** Metabolism, Vascular Biology & Angiogenesis

## Abstract

T. Haas and M. Rudnicki discuss the study by Wu et al, in this issue of *EMBO Mol Med*, that describes KRAS-dependent glycolytic reprogramming of endothelial cells in sporadic arteriovenous malformations.

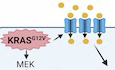

## Teaser

Brain arteriovenous malformations (bAVMs) are vascular anomalies characterized by a knot of intertwined and enlarged blood vessels. They form atypical direct connections (shunts) between the arterial and venous sides of the circulation, bypassing the capillary bed. This pathological flow pattern exposes fragile veins to excess mechanical stress, predisposing them to rupture. Although the incidence of bAVMs is low, their clinical impact is substantial as rupture results in intracranial hemorrhage, which often leads to severe neurological consequences including stroke (Samaniego et al, 2024).

Therapeutic management of bAVMs remains challenging. Surgical resection, the standard of care for intact bAVMs, carries significant risk and is often contraindicated due to lesion size, complexity, and anatomical location. Alternative endovascular and radiosurgical occlusive interventions likewise are associated with variable efficacy and potential complications (Samaniego et al, 2024). Despite decades of investigation, no pharmacological treatment has been approved for bAVM therapy. Thus, there is a substantive need to identify the molecular mechanisms that underlie AVM formation and maintenance, to better direct the selection of therapeutic targets.

Although most bAVMs arise as sporadic congenital lesions without a hereditary pattern, more than half harbor somatic activating Kirsten rat sarcoma (KRAS) mutations within endothelial cells (Nikolaev et al, 2018). The oncogene KRAS typically elicits uncontrolled proliferation and invasiveness in cancer cells and thus has been heavily interrogated as a therapeutic target. With the potential exception of a class of small-molecule inhibitors specific for the KRAS^G12C^ variant, KRAS has been viewed as an undruggable target, thus shifting the therapeutic focus to the blockade of downstream pathways (Huang et al, 2021). Like other oncogenes, KRAS signaling drives a metabolic shift to glycolysis, a phenomenon known as the Warburg effect, which sustains high cellular biosynthetic activity. This metabolic pathway has attracted attention for its potential to offer alternative druggable targets (Kerk et al, 2021).

In this issue of *EMBO Molecular Medicine*, Wu et al apply this cancer biology knowledge to tackle the question of whether endothelial glycolysis represents a viable therapeutic target in KRAS-driven bAVMs (Wu et al, 2026). Their team demonstrates that the KRAS^G12V^ activating mutation, the most common variant in bAVMs, is sufficient to impose a Warburg-like metabolic state in cultured human endothelial cells. This was evidenced by elevated glucose uptake (achieved by enhanced membrane localization of GLUT1 transporter) and lactate production. Extensive transcriptomic and proteomic profiling confirmed the upregulation of glycolytic pathway components in KRAS^G12V^ cells and pinpointed the enzyme hexokinase 2 (HK2) as a prominent downstream effector. Endothelial cells from KRAS^G12V^-positive human bAVM samples displayed more intense HK2 immunoreactivity than control samples, supporting the occurrence of this metabolic shift in the clinical condition.

Following on prior evidence that KRAS^G12V^ hyperactivates the mitogen-activated protein kinase (MAPK) ERK in endothelial cells (Fish et al, 2020), the researchers show that pharmacological inhibition of its upstream activator, MAPK kinase (MEK), suppresses glucose uptake and glycolytic flux in these cells. This finding indicates that glycolytic reprogramming can be driven by aberrant MAPK signaling.

**Figure 1:**
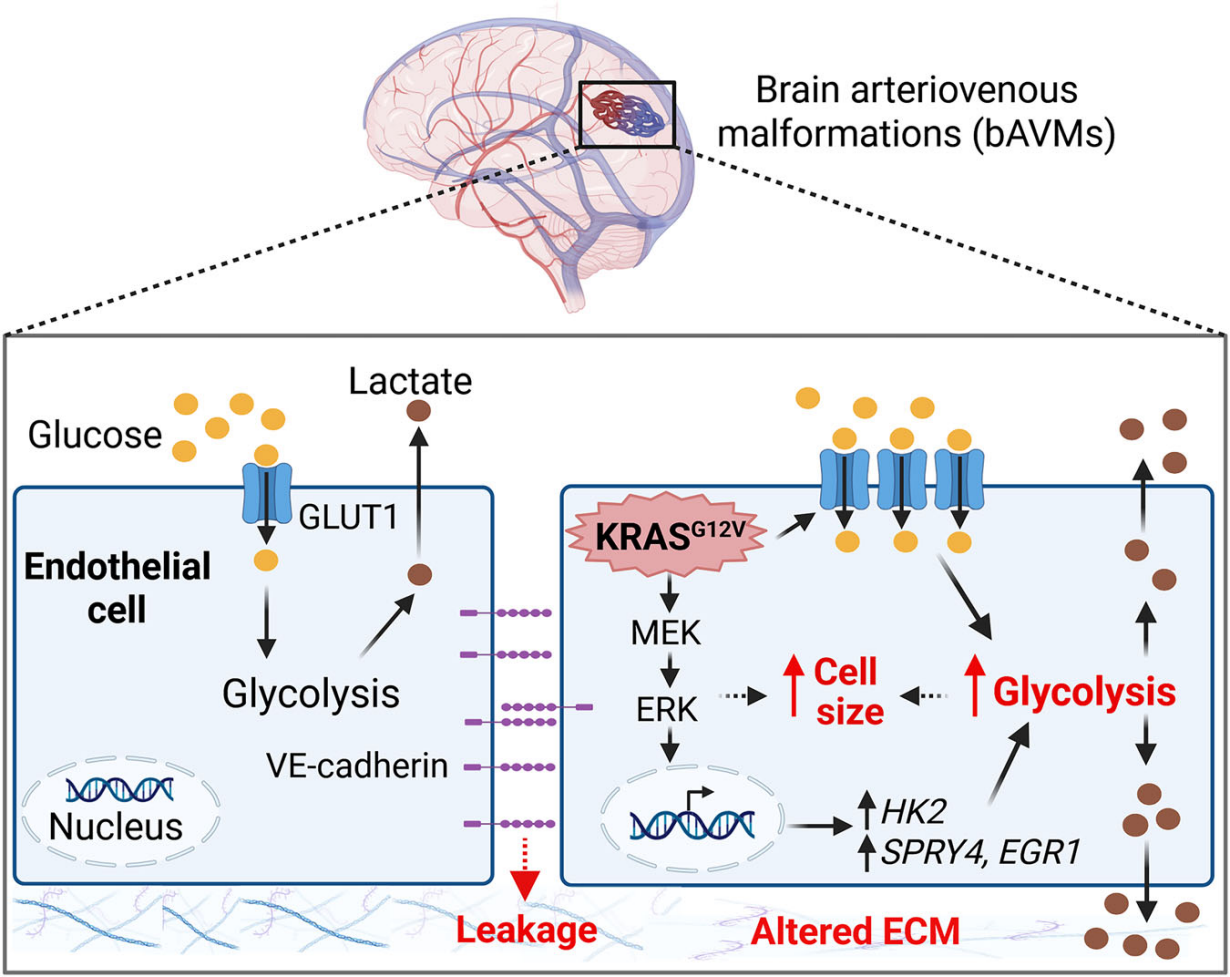
Somatic activating KRAS mutations, a major cause of sporadic bAVMs, profoundly alter endothelial cell biology. In this issue, Wu and colleagues establish that KRAS^G12V^ enhances glucose uptake and glycolysis in endothelial cells by increasing membrane localization of glucose transporters (GLUT1) and elevating hexokinase 2 (HK2) levels (Wu et al, 2026). They demonstrated that this occurs downstream of MAPK/ERK signaling, with concomitant upregulation of the canonical genes *SPRY4* and *EGR1*. Enhanced glycolysis supports cell enlargement, migration, and sprouting. In parallel, KRAS mutation alters extracellular matrix proteins and reduces membrane localization of VE-cadherin, increasing leakage and re-shaping the local environment. Schematic created in BioRender. Rudnicki M (2025) https://BioRender.com/kg903wa.

Phenotypically, KRAS^G12V^-positive endothelial cells exhibited a marked increase in cell size, decreased junctional vascular endothelial (VE)-cadherin, increased leakage, and enhanced migratory and sprouting activity, but no increase in proliferation (Fig. 1). As these aberrant behaviors may contribute to bAVM pathogenesis, the team tested the effects of glycolysis inhibition. Notably, treatment with 2-Deoxy-D-Glucose (2-DG), a non-metabolizable glucose mimetic that inhibits glycolytic flux, effectively reduced angiogenic sprouting and migration in KRAS^G12V^ cells, comparable to that previously observed with MEK inhibition (Fish et al, 2020).

To extend beyond the primary model of cultured endothelial cells, pharmacological inhibition of MAPK signaling and/or glycolysis was evaluated in a zebrafish model of AVM, in which injection of the KRAS^G12V^ mutant construct induces the formation of arteriovenous shunts. Inhibition of either MEK or glycolysis promoted partial rescue of shunt structures, while combined low-dose inhibition of MEK and the glucose transporter GLUT1 produced the most robust correction. This noteworthy outcome raises the possibility that therapeutic benefit might be achieved at reduced doses, potentially reducing undesired drug effects. Reinforcing the translational relevance of these findings, endothelial cells isolated from patient-derived bAVM samples also responded favorably to glycolysis inhibition on its own or in combination with MEK inhibition.

In summary, Wu and colleagues convincingly established that manipulation of glycolysis suppresses many pathological alterations in KRAS^G12V^ endothelial cells. A few considerations, however, need to be highlighted for future elucidation. The comparison of several small-molecule inhibitors of glycolysis revealed substantive non-overlapping effects on endothelial phenotype, indicating that further preclinical optimization of drug choice will be needed prior to clinical translation. Moreover, the KRAS^G12V^ variant was previously shown to disrupt other aspects of vascular organization, such as aberrant lumen expansion and impaired pericyte investment, in three-dimensional co-culture assays (Sun et al, 2022), emphasizing the importance of evaluating the influence of metabolic interventions in more comprehensive models that better mimic vessel structure. It will be crucial to evaluate the efficacy of glycolysis inhibitors using an in vivo mammalian bAVM model, potentially taking advantage of existing mouse models that recapitulate various forms of bAVMs (Ueki et al, 2025). This critical step will also address safety questions associated with sustained glycolytic inhibition in vivo, given the central role of glucose metabolism in normal tissues. In doing so, suppression of glycolysis as a generalized strategy for treatment of diverse types of AVMs can be investigated, as this could ease the need for a priori identification of genetic variants in patients.

Nonetheless, the current study significantly advances our knowledge of the molecular underpinnings of the endothelial cell phenotype characterizing KRAS^G12V^ brain AVMs. By identifying endothelial glycolysis as a modifiable component of KRAS-driven vascular malformations, Wu and colleagues persuasively argue that glycolysis-limiting drugs could augment the very limited arsenal of therapies available for patients with brain AVMs. Several inhibitors of glycolysis and MEK have already advanced through clinical trials for cancer indications; therefore, addressing the remaining mechanistic and translational questions could strengthen the rationale for rapid drug repurposing in bAVM treatment.
